# Supplementary material for: Ideonella sakaiensis Can Metabolize Bisphenol A as a Carbon Source
Source: Microorganisms. 2023 Nov 30;11(12):2891. doi: 10.3390/microorganisms11122891 (PMC10745525; doi:10.3390/microorganisms11122891)
Supplement: Supplementary file 1 [file microorganisms-11-02891-s001.zip › Supplementary Figure S1. growth 72h.pdf]

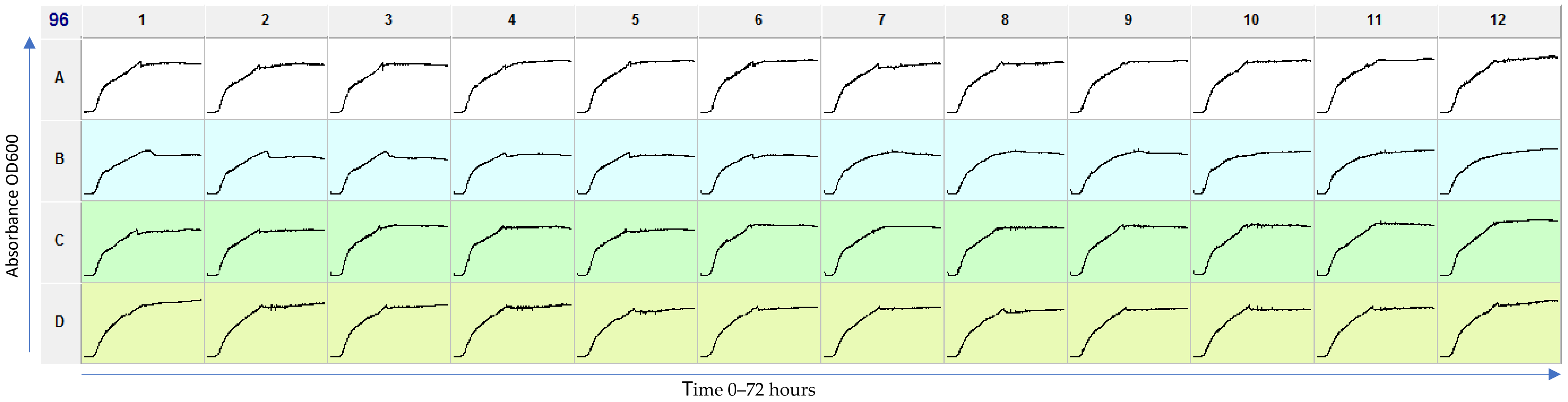

Supplementary figure 1. *I. sakaiensis* growth at 23 °C incubation. Group A represented by analites in EtOH: A1–3 BPA; A4–6 BPS; A7–12 Control; Group B represented by nutrient media at 70% concentration, analites in EtOH: B1–3 BPA; B4–6 BPS; B7–12 Control. Group C represented by Control with EtOH: C1–12. Group D represented by Control without EtOH: D1–12.
