# Supplementary material for: Ideonella sakaiensis Can Metabolize Bisphenol A as a Carbon Source
Source: Microorganisms. 2023 Nov 30;11(12):2891. doi: 10.3390/microorganisms11122891 (PMC10745525; doi:10.3390/microorganisms11122891)
Supplement: Supplementary file 1 [file microorganisms-11-02891-s001.zip › Supplementary Material S1.pdf]

The following models have been tried”

1. **Exponential Model**:

$$N(t) = N_0 e^{rt}$$

$N(t)$ is the number of bacteria at time t0, N_0 is the initial number of bacteria, e is Euler's number (approximately equal to 2.71828), and �r is the growth rate.

2. **Logistic Model**:

$$N(t) = \frac{K}{1 + \left(\frac{K - N_0}{N_0}\right) e^{-rt}}$$

- Where K is the carrying capacity, or the maximum population size that the environment can sustain indefinitely.
-

3. **Gompertz Model**:

$$N(t) = Ae^{-e^{\frac{ume}{A}(l-t)+1}}$$

- Where A is the upper asymptote, um is the maximum growth rate, e is Euler's number, and l is the lag time.

The results show that in many cases, the Gompertz model provides the best fit based on AIC values, indicating it captures the growth dynamics across the entire curve, including the lag, exponential, and stationary phases.

These references provide a detailed description of the models and their applications in microbiology and other biological sciences.

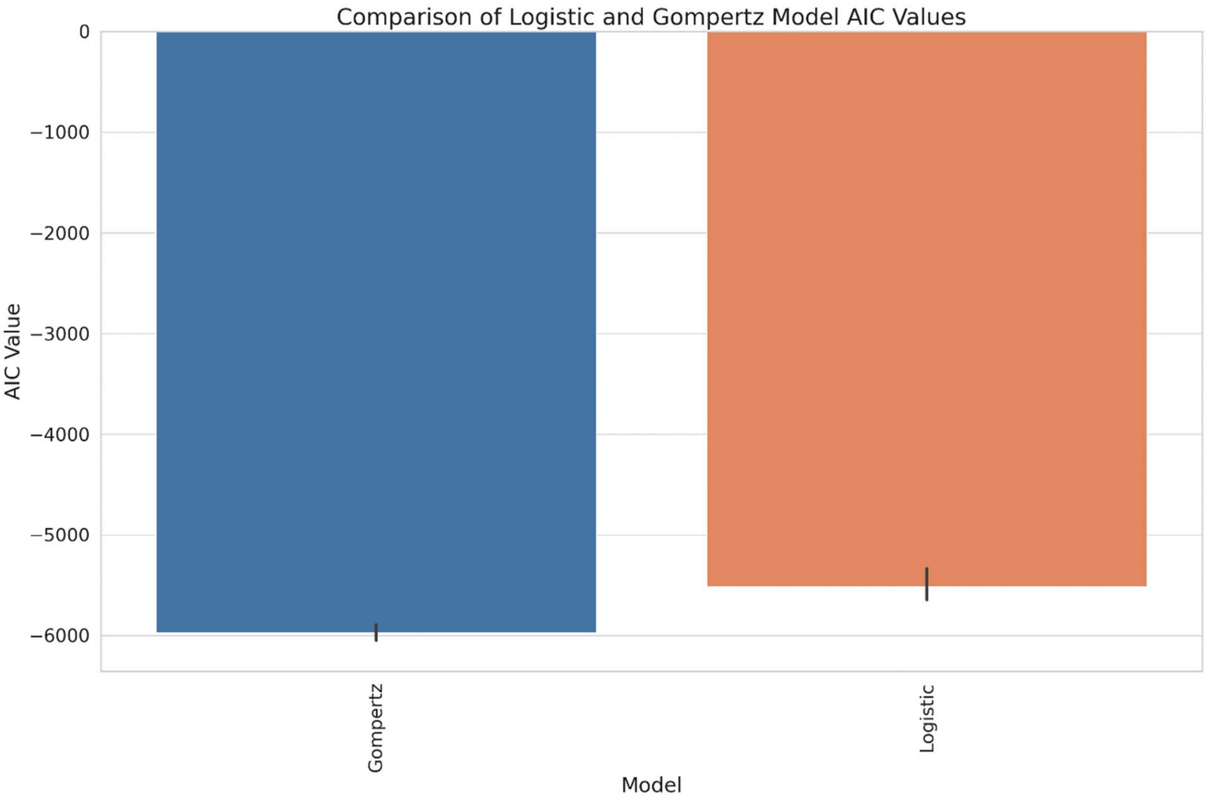

The representative fit for the growth curve of the condition labeled "control_1" using the best-fit model

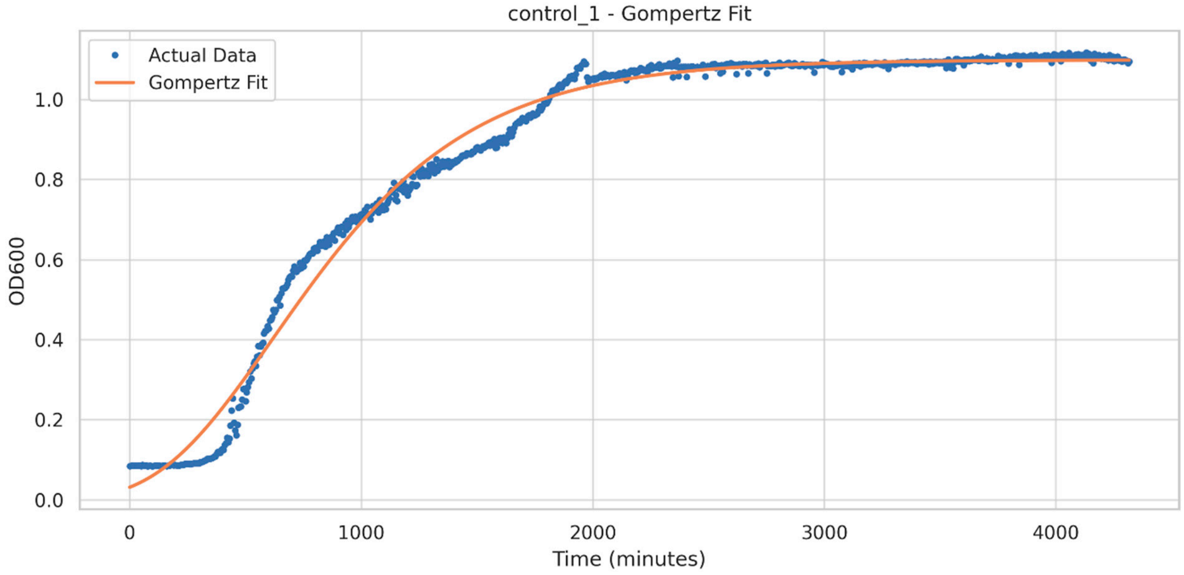

The descriptive statistics for the parameters of the Gompertz model across different conditions are as follows:

- **Upper Asymptote (A)**:
  - Mean: 1.0125
  - Standard Deviation (SD): 0.0971
  - Minimum: 0.8027
  - 25th Percentile: 0.9515
  - Median: 1.0356
  - 75th Percentile: 1.0808
  - Maximum: 1.1689
- **Maximum Growth Rate (um)**:
  - Mean: 0.0008
  - SD: 0.0001
  - Minimum: 0.0006
  - 25th Percentile: 0.0008
  - Median: 0.0008
  - 75th Percentile: 0.0008
  - Maximum: 0.0010
- **Lag Time (l)**:
  - Mean: 120.3 minutes
  - SD: 32.7 minutes
  - Minimum: 1.4 minutes
  - 25th Percentile: 110.6 minutes
  - Median: 120.6 minutes
  - 75th Percentile: 136.8 minutes
  - Maximum: 176.6 minutes

These statistics provide a summary of the central tendency and dispersion of the growth parameters across all conditions. Here are a few observations:

- The upper asymptote (A) is close to 1 for most conditions, suggesting a similar maximum optical density is reached by the bacteria across different conditions.
- The maximum growth rate (um) has a relatively low standard deviation, indicating that the rate at which the maximum growth occurs is quite consistent across conditions.
- There is considerable variability in the lag time (l), as evidenced by the standard deviation. This could indicate that different conditions affect the time it takes for bacteria to start the logarithmic growth phase.

The results of the tests to check the preconditions for ANOVA are as follows:

For the **Shapiro-Wilk test for normality**, which tests the null hypothesis that the data was drawn from a normal distribution

Maximum Growth Rate (um): p=0.6572. Since p>0.05, we fail to reject the null hypothesis, suggesting that the data may come from a normal distribution.

The corrected one-way ANOVA for the maximum growth rate (um) parameter across the different conditions (with replicates properly accounted for) yields the following results:

- **ANOVA Statistic (F-value)**: 1.03
- **p-value**: 0.40

Since the p-value is greater than 0.05, we fail to reject the null hypothesis of the ANOVA, which is that there are no significant differences in the maximum growth rate (um) across the different conditions.

This result suggests that the maximum growth rates are not statistically significantly different across the conditions tested, given the data available.

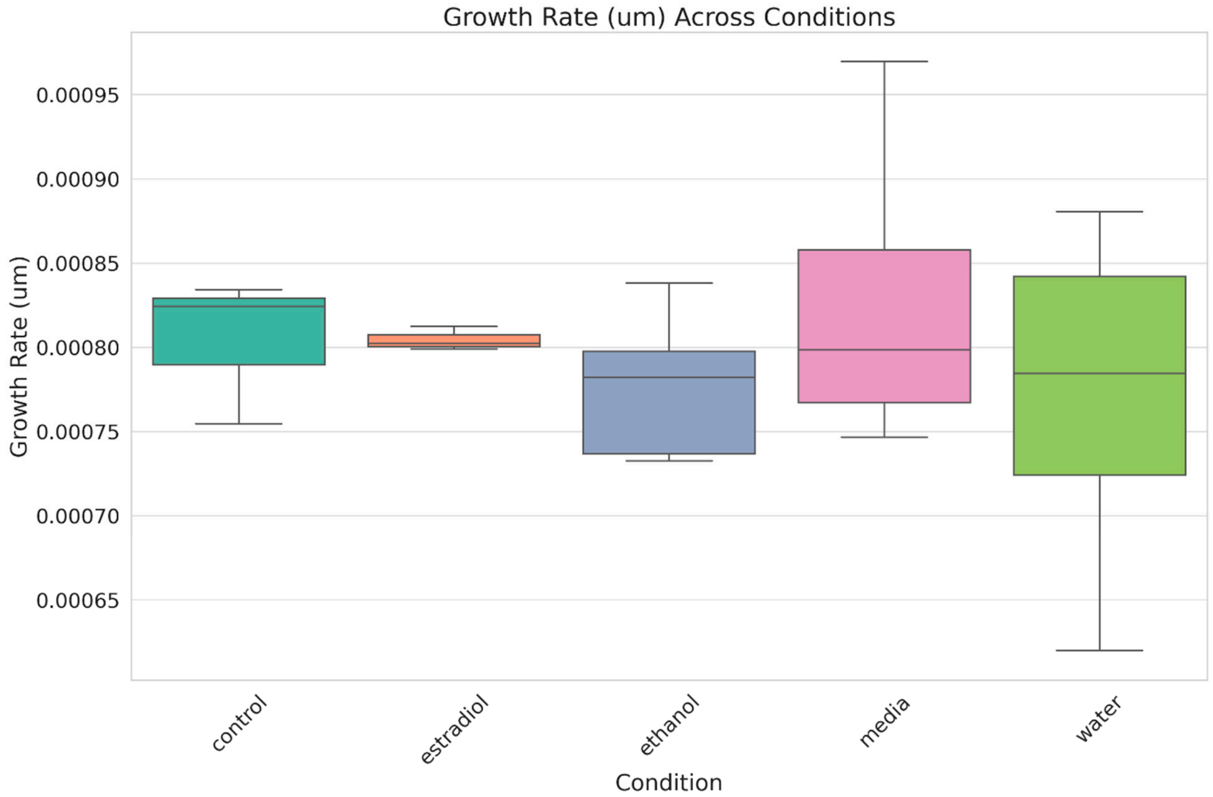

The boxplot with larger fonts, suitable for publication, has been created. You can download the high-resolution image using the following link:

Download Growth Rates Boxplot with Larger Fonts

The boxplot visualizing the growth rates (um) across different conditions

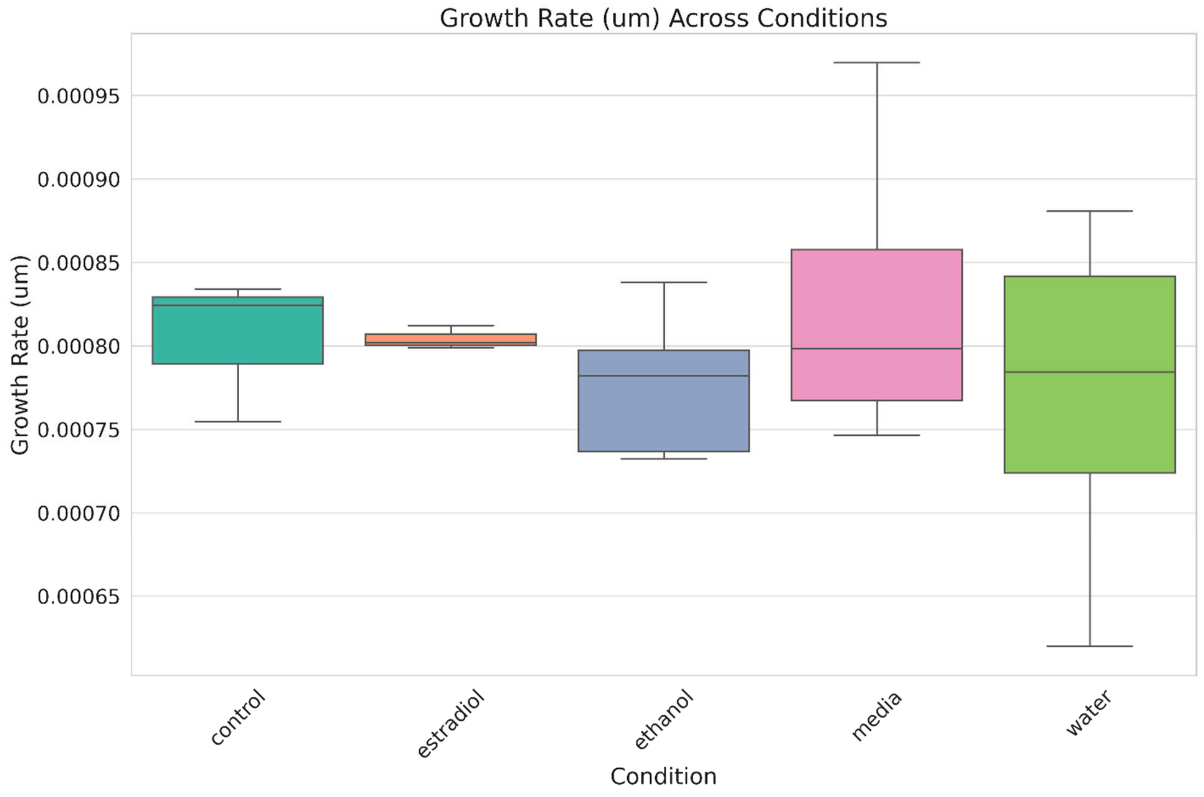

References

For references, these models are well-established in the literature. Here are some key references you can cite for each model:

- Zwietering, M.H., Jongenburger, I., Rombouts, F.M., van 't Riet, K., 1990. Modeling of the bacterial growth curve. Applied and Environmental Microbiology 56, 1875–1881.
- Winsor, C.P., 1932. The Gompertz curve as a growth curve. Proceedings of the National Academy of Sciences 18, 1–8.

Time to Reach Half Maximum (t_50):

- Calculate the time it takes for the bacterial culture to reach half of its maximum OD600 for each condition. This can be a useful comparative metric.

Here are the results after correctly grouping the data by replicates:

1. **Grouped t_{50} Data**: We have the mean, standard deviation, count, and standard error of the mean (SEM) for each base condition.
2. **Normality Test Results**:
   - Most conditions appear to have a non-significant p-value, indicating no violation of the normality assumption. However, there are two conditions with a p-value that's not a real number (likely due to a calculation issue because the standard deviation is zero, suggesting no variation in the data for `ethanol_BPS`). This might need further investigation or a different approach to normality testing.
3. **Levene's Test for Equal Variances**:
   - The Levene's test has a p-value of 0.1316, which is above the typical alpha level of 0.05, suggesting that we do not have sufficient evidence to reject the null hypothesis of equal variances across groups.

The one-way ANOVA test has been conducted, and it returned a statistic of approximately 6.08 with a very small p-value of approximately 2.12×10^{-5}. This p-value is below the common alpha threshold of 0.05, indicating that there are statistically significant differences in the t_{50} values among the different growth conditions.

The table of statistically significant differences from Tukey's HSD test

Statistically Significant Differences in t_50- Tukey HSD Test

group1	group2	meandiff	p-adj	lower	upper	reject
media	media_ethanol	-122.2222	0.001	-194.9474	-49.497	True
media	water_BPA	-125.0	0.0107	-231.4588	-18.5412	True

media	water_control	-148.3333	0.0013	-254.7921	-41.8746	True
media	water_BPS	-166.6667	0.001	-273.1254	-60.2079	True

The effect size analysis using Cohen's d for the pairs of conditions that showed statistically significant differences in the Tukey HSD post-hoc test has been calculated. Here are the results:

1. Comparison between `media` and `water_BPS`:
   - Cohen's d: 3.7184
   - Mean Difference: -166.6667
   - p-value: 0.0010
2. Comparison between `media` and `water_BPA`:
   - Cohen's d: 2.8571
   - Mean Difference: -125.0000
   - p-value: 0.0107
3. Comparison between `media` and `water_control`:
   - Cohen's d: 2.6290
   - Mean Difference: -148.3333
   - p-value: 0.0013
4. Comparison between `media` and `media_ethanol`:
   - Cohen's d: 2.1935
   - Mean Difference: -122.2222
   - p-value: 0.0010

These results indicate large effect sizes for all the comparisons, with the comparison between `media` and `water_BPS` having the largest effect size, followed by the comparisons with `water_BPA` and `water_control`. The negative mean differences indicate that the t_50 values for the `media` condition are lower than those for the other conditions, suggesting faster growth in the `media` condition.
